# VeloxChem: Large-Scale DFT Calculations of Geometric Derivatives up to Second Order for Simulation of IR Spectra

**DOI:** 10.1021/acs.jpca.5c04510

**Published:** 2026-01-06

**Authors:** Josefine H. Andersen, Iulia Emilia Brumboiu, Manuel Hodecker, Xin Li, Patrick Norman, Zilvinas Rinkevicius

**Affiliations:** † Division of Theoretical Chemistry and Biology, School of Engineering Sciences in Chemistry, Biotechnology and Health, 7655 KTH Royal Institute of Technology, SE-100 44 Stockholm, Sweden; ‡ Faculty of Physics, Astronomy and Informatics, 49577 Nicolaus Copernicus University in Toruń, 87-100 Toruń, Poland; § PDC Center for High Performance Computing, 7655 KTH Royal Institute of Technology, SE-100 44 Stockholm, Sweden

## Abstract

A software implementation of analytic geometric derivatives of electron-repulsion integrals up to second order is presented for the modeling of vibrational spectroscopies at the level of first-principles Kohn–Sham density functional theory (DFT). In line with the general goals of the VeloxChem program, it targets efficient execution in high-performance computing environments with a hybrid MPI/OpenMP parallelization model and is based on the technique of automatic C++ code generation for high versatility. Gradient calculations scale identically with conventional Fock matrix constructions, and also with the prefactor taken into account, the computational cost of the gradient is significantly lower than that of the self-consistent field (SCF) optimization of the reference state. The Hessian calculation shows a scaling of *N* ^3.5^ with *N* being the number of contracted Gaussian basis functions. The computational bottleneck in the Hessian calculation is the solving of the coupled-perturbed Kohn–Sham equations that with VeloxChem can be offloaded to GPU-accelerated nodes. The large-scale virtues of the presented software module are demonstrated by the DFT/B3LYP calculation of the IR spectrum of the entire ubiquitin protein with 1,152 atoms in the quantum mechanical (QM) region and TIP3P water in the molecular mechanics (MM) region. The simulated amide I band shows to be in excellent agreement with experiment.

## Graphical abstract

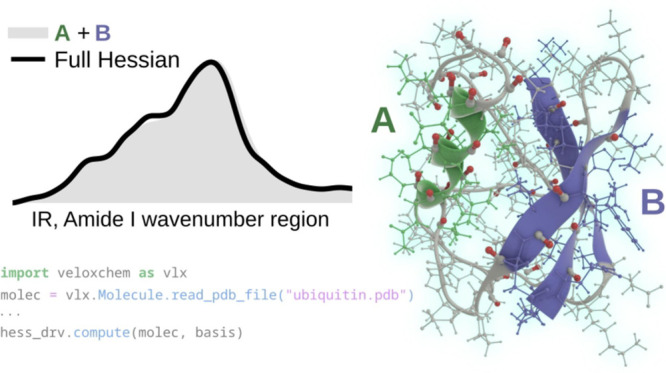

## Introduction

Over the years, a plethora of spectroscopies have been developed and employed for the characterization of molecular compounds and materials.^1^ In the present work, we will be concerned with infrared (IR) absorption spectroscopy that exists in various forms designed for different intents and purposes such as e.g. surface-enhanced IR spectroscopy for the detection of minute amounts of compounds in sensor applications,^2^ atomic force microscopy-based IR spectroscopy to reach a spatial resolution far below the optical diffraction limit,^3^ Fourier-transform IR spectroscopy for the characterization of heterogeneous catalytic reactions,^4^ and IR difference spectroscopy for the time-resolved detection of protonation and H-bonding changes in proteins.^5^ Applications of IR spectroscopies vary broadly from material science with studies of systems as complex as guest molecules in metal–organic frameworks^6^ to life sciences with studies of proteins in complex bioenvironments.^7–11^

Owing to the sensitiveness toward hydrogen bonding, IR absorption spectroscopy is a powerful approach for the study of protein structures.^12–16^ Nine characteristic bands arise from the absorption of IR radiation by proteins namely the amide A, B, and I–VII bands. Out of these, the amide I band found in the frequency region of 1700–1600 cm^–1^ is the most sensitive toward the secondary structures of proteins. The underlying vibrational modes of this band are almost entirely due to C=O stretching vibrations, and while hardly being affected by the amino acid side chains, these modes are sensitive to the neighboring amide groups in the peptide backbone as well as the hydrogen bonding patterns that, in turn, differ among the secondary structures, most notably α-helices and β-sheets.

In 2020, Baronio and Barth concluded:^17^

The amide I region of the infrared spectrum is related to the protein backbone conformation and can provide important structural information. However, the interpretation of the experimental results is hampered because the theoretical description of the amide I spectrum is still under development.

The authors referred to the high computational cost involved with density functional theory (DFT) calculations of IR spectra which, at the time, had limited such a first-principles approach to small peptide systems.^18–25^ Targeting proteins, a transition dipole coupling (TDC) model with parameters optimized for the major secondary structures was instead proposed.^17^ The TDC approach^17,26,27^ is not without alternatives, however, and other works have adopted force fields,^18,28^ empirical modeling,^29^ and the semiempirical density functional tight-binding (DFTB) approach coupled with the fluctuating charges force field.^30^

Methods for simulations of IR spectra based on first-principles methods have been available for a long time, also in combination with molecular mechanics (MM) and polarizable embedding environments to construct various forms of QM/MM approaches.^31–39^ On the more technical side of things, computational strategies to reach linear scaling have been adopted for the calculation of the Hessian and demonstrating application to systems with close to 300 atoms.^40^ Other ways to reach larger system size is to adopt some fragmentation scheme and partition the system into subsystems. We here note the fragment molecular orbital method for which analytic second-order derivatives have been developed and implemented^41–43^ and the partial Hessian vibrational analysis approach in which a subblock of the Hessian matrix is diagonalized.^44,45^

In the present work, we present an implementation of analytic Hessians and IR spectra at the DFT level of theory in the VeloxChem program^46,47^ and demonstrate its large-scale capabilities. In line with the overarching goals of the VeloxChem initiative, the software design targets execution in high-performance computing (HPC) environments where massively parallel computations are made available on multicore CPUs and top-tier GPUs.^47^ VeloxChem leverages such supercomputers by means of a hybrid programming language model with an upper Python code layer that is hardware aware and handles all the MPI communication and a lower C++/CUDA/HIP code layer that handles all the threaded parallelization. Compared to the original program presentation in 2020,^46^ we have written a completely new CPU code based on automatic code generation for the calculation of electron-repulsion integrals (ERIs) and extended it to encompass first- (gradients) and second-order (Hessians) geometric derivatives. The 3*N* (with *N* referring to the number of atoms in the system) coupled-perturbed Kohn–Sham (CPKS) equations that must be solved in the calculation of the Hessian can be off-loaded to GPUs by making use of the previously presented GPU linear response solver module,^47^ and we have here employed this option to reduce wall times in the simulations. With a study of the entire ubiquitin protein in aqueous solution, we illustrate how large-scale IR spectrum calculations at the DFT level are made readily available with use of HPC resources. No other numerical “trick” than the conventional (and, in our case, conservative) integral screening based on the Cauchy–Schwarz inequality is employed, so our example should be indicative of size limits in the general case. Ubiquitin has secondary structures of both α-helix and β-sheet character and our analysis contains an assessment of the partial Hessian approximation where the two types of secondary structures are split into separate subsystems.

In the next section, we detail our implementation of the ERIs and exchange-correlation kernel integration, both based on well-established algorithms, underlying our efficient calculations of analytic Hessians, and the associated code performance and scaling are also presented. In the following section, we introduce our example application and present the IR spectrum simulation of ubiquitin with a comparison made against experiment for the amide I band that represents the signature band for secondary structures in proteins.

## Implementation

### Evaluation of Electron-Repulsion Integrals

In first-principles quantum chemistry methods, the evaluation of ERIs is the most time-consuming step. To a large degree, the ability to rapidly compute ERIs determines the practical limits in applicability of the various quantum mechanical (QM) methods, and most quantum chemistry programs are built around specific implementations of ERI evaluation drivers. The VeloxChem program implements the ERIs evaluation engine based on a modified Obara–Saika scheme that combines the vertical Obara–Saika recursions^48^ (VRRs) with horizontal transfer recursions^49^ (HRRs). This engine is optimized for segmented basis sets, such as the def2 family of basis sets developed by Weigend and Ahlrichs.^50^

In this work, we extend the VeloxChem ERI-engine to encompass the calculation of first- and second-order geometrical derivatives of ERIs. Before discussing the implementation details of this extension, let us briefly review the structure of the VeloxChem scheme for the evaluation of regular ERIs shown in Figure 1. In this scheme, the ERIs are given in shell-quartet form

$$(\mathrm{ij}|\mathrm{kl})=\iint \chi_{i}(r,A)\chi_{j}(r,B)\frac{1}{\left|r-r^{\prime }\right|}\chi_{k}(r^{\prime },C)\chi_{l}(r^{\prime },D)d^{3}rd^{3}r^{\prime }$$ 1

and they are computed in nine steps, where the last step involves processing the integral by distributing its components into the Fock matrix. The adopted strategy leverages an early contraction of primitive Cartesian ERIs (step four in Figure 1) and an early transformation of the contracted Cartesian ERIs into mixed Cartesian/spherical form (step six in Figure 1) to minimize the overall computational cost. Here, ***i***, ***j***, ***k***, and ***l*** represent a set of spherical angular momentum components forming a shell-quartet of the contracted spherical Gaussian functions, χ, centered on atomic positions **A**, **B**, **C**, and **D**, respectively.

**Figure 1:**
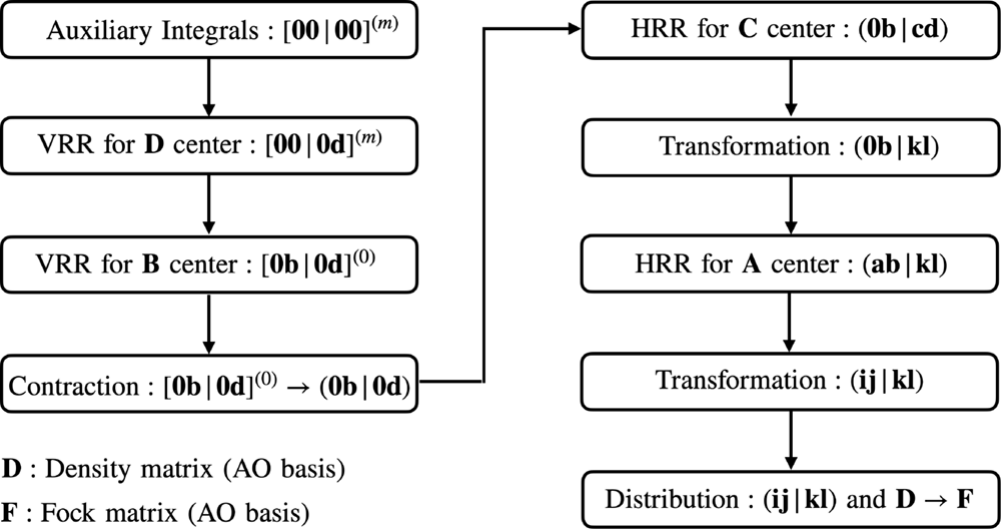
Evaluation scheme for ERIs in VeloxChem. The first three steps are carried out in the primitive Cartesian basis, where [**00**|**00**]^(*m*)^ (*m* = 0...|**i**| + |**j**| + |**k**| + |**l**|) is found in eq 2, [**00**|**0d**]^(*m*)^ (**d** = **k**...**k** + **l**, *m* = 0...|**i**| + |**j**|) is computed using eq 3, and [**0b**|**0d**]^(0)^ (**b** = **i**...**i** + **j**, **d** = **k**...**k** + **l**) is computed using eq 4. The remaining recurrence relations are applied in contracted Cartesian Gaussian basis or contracted mixed Cartesian/spherical Gaussian basis, where (**0b**|**cd**) (**b** = **i**...**i** + **j**, **d** = **k**, **d** = **l**) is computed using eq 5, and (**ab**|**kl**) (**a** = **i**, **b** = **j**) is computed using eq 6.

The first three steps in the presented ERIs evaluation scheme are carried out in the primitive Cartesian Gaussian basis, where the first step is the computation of auxiliary integral shell-quartets, and the remaining two steps are the application of the VRRs to atoms **B** and **D** to generate the [**0c**|**0d**]^(0)^ shell-quartets required by the HRRs. More specifically, the calculation of the auxiliary integrals involves the evaluation of pairwise overlaps *S*_AB_ and *S*_CD_ as well as the Boys function *F*_m_(*T*) according to

$$\left[00|00{]}^{\left(m\right)}=2{\left(\frac{\rho }{\pi }\right)}^{1/2}S_{\mathrm{AB}}S_{\mathrm{CD}}F_{m}(T\right)$$ 2

Here, *S*_AB_ and *S*_CD_ are computed using^48^ and *F*_m_(*T*) is computed using eqs 45 and 46 in the original work of Obara and Saika.^48^

The second step in the ERI evaluation is the generation of the primitive [**00**|**0d**]­(*m* ^′^) shell-quartets via a VRR applied to atom **D**. The angular momentum on the basis function associated with atom **D** can be raised to the required angular momentum values by repeated employment of the four-term VRR according to

$$[00|0d+1_{i}{]}^{\left(m\right)}={QD}_{i}[00|0d{]}^{\left(m\right)}+{WQ}_{i}[00|0d{]}^{\left(m+1\right)}+\frac{N_{i}(d)}{2\eta }[00|0d-1_{i}{]}^{\left(m\right)}-\frac{N_{i}(d)\rho }{2\eta^{2}}[00|0d-1_{i}{]}^{\left(m+1\right)}$$ 3

where we have introduced the shorthand notation for distance components, **AB**_*i*_ = **A**_*i*_ – **B**_*i*_, and adopted the definition of quantities entering the recurrence relations from the original Obara and Saika work.^48^

Similarly to the second step, the third step in the ERIs evaluation amounts to the generation of primitive [**0b**|**0d**]^(0)^ shell-quartets from the primitive [**00**|**0d**]^(*m′*)^ shell-quartets by applying a VRR to atom **B**. The VRR in this third step is slightly more complicated compared to the second step and contains five terms

$$[0b+1_{i}|0d{]}^{\left(m\right)}={PB}_{i}[0b|0d{]}^{\left(m\right)}+{WP}_{i}[0b|0d{]}^{\left(m+1\right)}+\frac{N_{i}(b)}{2\zeta }[0b-1_{i}|0d{]}^{\left(m\right)}-\frac{N_{i}(b)\rho }{2\zeta^{2}}[0b-1_{i}|0d{]}^{\left(m+1\right)}+\frac{N_{i}(d)}{2(\zeta +\eta )}[0b|0d-1_{i}{]}^{\left(m+1\right)}$$ 4

The remaining recurrence steps in the ERIs evaluation scheme (see Figure 1) are HRRs, which are dependent only on distances between atoms and thus can be carried out over contracted integral shell-quartets. Thus, in the fourth step, all required primitive integral shell-quartets are transformed into contracted integral shell-quartets. Following this step, the HRR on atom **C** is applied to generate (**0b**|**cd** *′*) shell-quartets (fifth step in Figure 1). This recurrence relation is trivial and involves only two terms

$$\left(0b|c+1_{i}d)={CD}_{i}(0b|cd)+(0b|cd+1_{i}\right)$$ 5

In the next two steps, we exploit the fact that the HRRs for atoms **A** and **C** are independent, and thus, HRR for the **A** atom can be carried out using contracted integral shell-quartets in a mixed representation. Taking this into account, we first transform the ket side of the (**0b**|**cd** *′*) shell-quartets from Cartesian representation to spherical representation, thereby substantially reducing the number of applications of the HRR to compute the (**ab**|**kl**) integrals in the seventh step. The HRR for atom **A** is very similar to the HRR for atom **C**, the only difference being that it operates on integral shell-quartets in mixed representations, i.e.

$$\left(a+1_{i}b|kl)={BA}_{i}(ab|kl)+(ab+1_{i}|kl\right)$$ 6

The final two steps in the ERI evaluation scheme are more to be seen as management, including the transformation of integrals from mixed to spherical representation and the distribution of transformed integrals into the Fock matrix.

The described ERI scheme is readily extended to handle geometric derivatives in a manner that similarly leverages an early contraction of integrals to minimize the overall computational cost. More specifically, the computation of geometrical derivatives of ERIs requires one additional VRR to generate geometrical derivatives of primitive ERIs and the replacement of appropriate HRRs with differentiated HRRs. Since calculations of various second-order geometrical derivatives of ERIs are very similar, we will illustrate the implementation details with second-order geometrical derivatives of ERIs with respect to the single atomic center. This scheme is presented in Figure 2.

**Figure 2:**
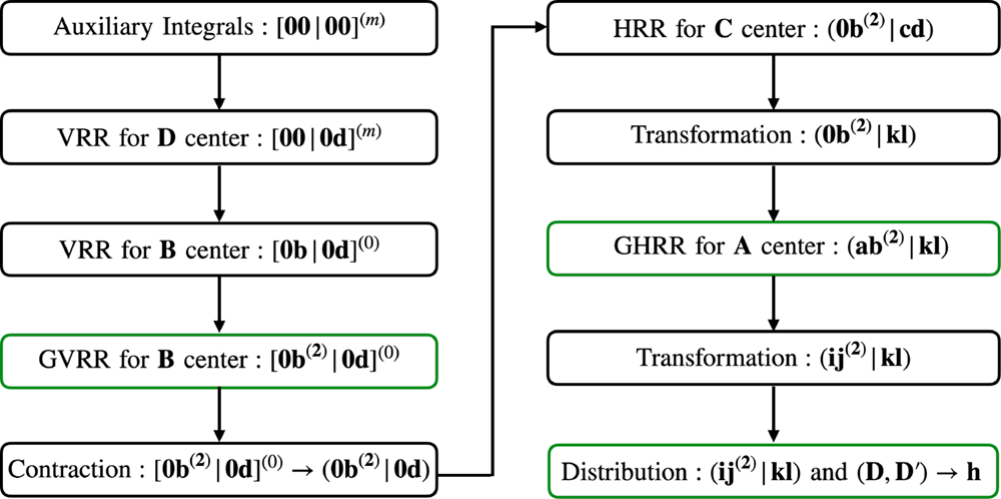
Evaluation scheme for ERI Hessian on center **B** in VeloxChem. The first four steps are carried out in the primitive Cartesian basis, where [**00**|**00**]^(*m*)^ (*m* = 0...|**i**| + |**j**| + |**k**| + |**l**| + 1) is found in eq 2, [**00**|**0d**]^(*m*)^ (**d** = **k**...**k** + **l**, *m* = 0...|**i**| + |**j**| + 1) is computed using eq 3, [**0b**|**0d**]^(0)^ (**b** = **i**...**i** + **j** + **1**, **d** = **k**...**k** + **l**) is computed using eq 4, and [**0b** ^**(2)**^|**0d**]^(0)^ (**b** = **i**...**i** + **j**, **d** = **k**...**k** + **l**) is computed using eq 7. The remaining recurrence relations are applied in contracted Cartesian Gaussian basis or contracted mixed Cartesian/spherical Gaussian basis, where (**0b** ^**(2)**^|**cd**) (**b** = **i**...**i** + **j**, **d** = **k**, **d** = **l**) is computed using eq 5, and (**ab** ^(2)^|**kl**) (**a** = **i**, **b** = **j**) is computed using eq 8. The steps unique to geometrical derivatives are highlighted in green.

The first three steps in the computation of shell-quartet second-order geometrical derivatives are similar to the evaluation of the ERIs themselves and consist of the computation of auxiliary integrals followed by the application of VRRs on atoms **D** and **B**.

In the fourth step, the second-order geometrical derivatives of primitive ERIs is computed by applying recurrence relation for differentiation of the primitive Cartesian basis function with respect to its atomic center (GVRR),^48^ i.e.

$$\frac{\partial^{2}}{\partial B_{i}\partial B_{j}}[0b|0d{]}^{\left(0\right)}=[{0b}^{i,j}|0d{]}^{\left(0\right)}=4\zeta_{b}^{2}[0b+2_{i,j}|0d{]}^{\left(0\right)}-2\zeta_{b}(N_{j}(b)[0b+1_{i}-1_{j}|0d{]}^{\left(0\right)}+N_{i}(b)[0b+1_{j}-1_{i}|0d{]}^{\left(0\right)})-N_{j}(b)N_{i}(b)[0b-2_{i,j}|0d{]}^{\left(0\right)}$$ 7

This recurrence relation is independent of the differentiation center and can be applied to form any targeted geometrical derivative of ERIs.

Following the fourth step, the computation of geometrical derivatives of ERIs proceeds in the same fashion as the computation of the ERIs themselves. The only difference between the two procedures is the substitution of HRRs with their differentiated variants (GHRRs) in cases where HRRs are applied to the atoms with respect to which the geometrical derivative is taken. The required GHRRs are obtained by taking partial derivatives of eqs 5 or 6 with respect to the appropriate atoms. To illustrate the construction of GHRRs we consider the case of the second-order geometrical derivatives of ERIs with respect to atom **B**

$$\left(a+1_{i}b^{j,j^{\prime }}|kl)={BA}_{i}({ab}^{j,j^{\prime }}|kl)+(a(b+1_{i}{)}^{j,j^{\prime }}|kl)+\delta_{ij}({ab}^{j^{\prime }}|kl)+\delta_{ij^{\prime }}({ab}^{j}|kl\right)$$ 8

where the expression is obtained by taking a partial derivative with respect to the B atom position of the HRR given in eq 6.

Finally, the integrals distribution step in the evaluation scheme (see Figure 2) is slightly different from the normal ERIs distribution step. Instead of computing the Fock matrix by contracting integrals with the density matrix, the contribution to the molecular gradient from two-electron-repulsion integrals is directly computed via double contraction of gradient ERIs with two density matrices. The scheme described above for computing the second-order geometrical derivative of ERIs is directly transferable to higher-order geometrical derivatives of ERIs. It has been successfully implemented in the VeloxChem program to compute all geometrical derivatives of ERIs required for computing the molecular Hessian.

To conclude the discussion of our extension to the ERIs evaluation driver in the VeloxChem program, we point out that the code implementation for computing first- and second-order geometrical derivatives of ERIs follows a well-established hybrid OpenMP/MPI parallelization strategy,^46^ enabling highly efficient calculations on HPC systems of varying sizes. Furthermore, all VRRs and HRRs evaluation kernels have been optimized to exploit SIMD instructions on various CPUs, enabling rapid evaluation of these recursions. These algorithmic choices in our implementation has paved the way for the large-scale molecular Hessian calculations presented in this work.

### Exchange–Correlation Numerical Integration

Hessian calculations at the DFT level require the evaluation of two additional exchange–correlation (XC) terms apart from the regular XC contribution to the Fock matrix. The first one is the second-order partial derivative of the XC energy with respect to the displacement of the nuclei. For a closed-shell system and a density functional at the generalized gradient approximation (GGA) level, the second-order derivative can be written as^51–53^

$$\frac{\partial^{2}E_{\mathrm{xc}}}{\partial \xi \partial \zeta }=2\frac{\partial }{\partial \zeta }\int \left[f_{\rho_{\alpha }}\rho_{\alpha }^{\left(\xi \right)}+(2f_{\sigma_{\alpha \alpha }}+f_{\sigma_{\alpha \beta }})\nabla \rho_{\alpha }\cdot (\nabla \rho_{\alpha }{)}^{\left(\xi \right)}\right]d^{3}r$$ 9

where ρ and σ denote the density and the density gradient dot product as in GGA, *f* with its subscripts denotes functional derivative and ξ and ζ denote the displacement of the nuclei. Applying the chain rule to the above integrand results in

$$\frac{\partial }{\partial \zeta }[f_{\rho_{\alpha }}\rho_{\alpha }^{\left(\xi \right)}]=(f_{\rho_{\alpha }\rho_{\alpha }}+f_{\rho_{\alpha }\rho_{\beta }})\rho_{\alpha }^{\left(\xi \right)}\rho_{\alpha }^{\left(\zeta \right)}+(f_{\rho_{\alpha }\sigma_{\alpha \alpha }}+f_{\rho_{\alpha }\sigma_{\alpha \beta }}+f_{\rho_{\alpha }\sigma_{\beta \beta }})\rho_{\alpha }^{\left(\xi \right)}\sigma_{\alpha \alpha }^{\left(\zeta \right)}+f_{\rho_{\alpha }}\rho_{\alpha }^{\left(\xi ,\zeta \right)}$$ 10

and

$$\frac{\partial }{\partial \zeta }\left[\left(2f_{\sigma_{\alpha \alpha }}+f_{\sigma_{\alpha \beta }}\right)\nabla \rho_{\alpha }\cdot {\left(\nabla \rho_{\alpha }\right)}^{\left(\xi \right)}\right]=\left(2f_{\rho_{\alpha }\sigma_{\alpha \alpha }}+f_{\rho_{\alpha }\sigma_{\alpha \beta }}+2f_{\rho_{\beta }\sigma_{\alpha \alpha }}+f_{\rho_{\beta }\sigma_{\alpha \beta }}\right)\left(\nabla \rho_{\alpha }\cdot {\left(\nabla \rho_{\alpha }\right)}^{\left(\xi \right)}\right)\rho_{\alpha }^{\left(\zeta \right)}+\left(2f_{\sigma_{\alpha \alpha }\sigma_{\alpha \alpha }}+f_{\sigma_{\alpha \alpha }\sigma_{\alpha \beta }}+2f_{\sigma_{\alpha \alpha }\sigma_{\alpha \beta }}+f_{\sigma_{\alpha \beta }\sigma_{\alpha \beta }}+2f_{\sigma_{\alpha \alpha }\sigma_{\beta \beta }}+f_{\sigma_{\alpha \beta }\sigma_{\beta \beta }}\right)\left(\nabla \rho_{\alpha }\cdot {\left(\nabla \rho_{\alpha }\right)}^{\left(\xi \right)}\right)\sigma_{\alpha \alpha }^{\left(\zeta \right)}+\left(2f_{\sigma_{\alpha \alpha }}+f_{\sigma_{\alpha \beta }}\right){\left(\nabla \rho_{\alpha }\right)}^{\left(\xi \right)}\cdot {\left(\nabla \rho_{\alpha }\right)}^{\left(\zeta \right)}+\left(2f_{\sigma_{\alpha \alpha }}+f_{\sigma_{\alpha \beta }}\right)\nabla \rho_{\alpha }\cdot {\left(\nabla \rho_{\alpha }\right)}^{\left(\xi ,\zeta \right)}$$ 11

The other XC term that is needed for the DFT Hessian is the first-order partial derivative of the XC matrix element with respect to the displacement of the nuclei, which contributes to the right-hand side of the coupled-perturbed Kohn–Sham (CPKS) equations.^52,53^ It can be written as follows for a closed-shell system with a GGA functional

$$\frac{\partial }{\partial \xi }v_{\mu \nu }^{\mathrm{xc},\alpha }=\frac{\partial }{\partial \xi }\int [f_{\rho_{\alpha }}\chi_{\mu }\chi_{\nu }+(2f_{\sigma_{\alpha \alpha }}+f_{\sigma_{\alpha \beta }})\nabla \rho_{\alpha }\cdot \nabla (\chi_{\mu }\chi_{\nu })]d^{3}r$$ 12

where χ_μ_ and χ_ν_ denote atomic orbitals. Applying the chain rule to the integrand gives

$$\frac{\partial }{\partial \xi }\left(f_{\rho_{\alpha }}\chi_{\mu }\chi_{\nu }\right)=\left(f_{\rho_{\alpha }\rho_{\alpha }}+f_{\rho_{\alpha }\rho_{\beta }}\right)\chi_{\mu }\chi_{\nu }\rho_{\alpha }^{\left(\xi \right)}+\left(f_{\rho_{\alpha }\sigma_{\alpha \alpha }}+f_{\rho_{\alpha }\sigma_{\alpha \beta }}+f_{\rho_{\alpha }\sigma_{\beta \beta }}\right)\chi_{\mu }\chi_{\nu }\sigma_{\alpha \alpha }^{\left(\xi \right)}+f_{\rho_{\alpha }}\left(\chi_{\mu }^{\left(\xi \right)}\chi_{\nu }+\chi_{\mu }\chi_{\nu }^{\left(\xi \right)}\right)$$ 13

and

$$\frac{\partial }{\partial \xi }\left[\left(2f_{\sigma_{\alpha \alpha }}+f_{\sigma_{\alpha \beta }}\right)\nabla \rho_{\alpha }\cdot \nabla \left(\chi_{\mu }\chi_{\nu }\right)\right]=\left(2f_{\rho_{\alpha }\sigma_{\alpha \alpha }}+f_{\rho_{\alpha }\sigma_{\alpha \beta }}+2f_{\rho_{\beta }\sigma_{\alpha \alpha }}+f_{\rho_{\beta }\sigma_{\alpha \beta }}\right)\left(\nabla \rho_{\alpha }\cdot \nabla \left(\chi_{\mu }\chi_{\nu }\right)\right)\rho_{\alpha }^{\left(\xi \right)}+\left(2f_{\sigma_{\alpha \alpha }\sigma_{\alpha \alpha }}+f_{\sigma_{\alpha \alpha }\sigma_{\alpha \beta }}+2f_{\sigma_{\alpha \alpha }\sigma_{\alpha \beta }}+f_{\sigma_{\alpha \beta }\sigma_{\alpha \beta }}+2f_{\sigma_{\alpha \alpha }\sigma_{\beta \beta }}+f_{\sigma_{\alpha \beta }\sigma_{\beta \beta }}\right)\left(\nabla \rho_{\alpha }\cdot \nabla \left(\chi_{\mu }\chi_{\nu }\right)\right)\sigma_{\alpha \alpha }^{\left(\xi \right)}+\left(2f_{\sigma_{\alpha \alpha }}+f_{\sigma_{\alpha \beta }}\right){\left(\nabla \rho_{\alpha }\right)}^{\left(\xi \right)}\cdot \nabla \left(\chi_{\mu }\chi_{\nu }\right)+\left(2f_{\sigma_{\alpha \alpha }}+f_{\sigma_{\alpha \beta }}\right)\nabla \rho_{\alpha }\cdot {\left(\nabla \left(\chi_{\mu }\chi_{\nu }\right)\right)}^{\left(\xi \right)}$$ 14

These DFT contributions have been added to the XC kernel integration routines in the C++ layer of VeloxChem. The computational cost associated with the DFT contributions is small in comparison with the cost associated with the evaluation of ERIs and their derivatives, and it is for that reason left out from further discussion.

### Coupled-Perturbed Hartree–Fock/Kohn–Sham Equations

Molecular Hessians require the computation of perturbed molecular orbital (MO) coefficients (*C*_μ*i*_^ξ^), i.e., geometric derivatives of the reference MO coefficients (*C*_μ*p*_) with respect to nuclear coordinates, ξ. Starting from a linear ansatz in terms of the unperturbed coefficients^54^

$$C_{\mu i}^{\xi }=\sum_{p}C_{\mu p}U_{qi}^{\xi }$$ 15

the occupied-virtual block of the unknown **U** ^ξ^ matrix requires solving a set of coupled-perturbed Hartree–Fock/Kohn–Sham (CPHF/CPKS) equations. For a closed-shell molecule, the CPHF/CPKS equations take the form^53^

$$(\epsilon_{i}-\epsilon_{a})U_{ai}^{\xi }-2G_{ai}[U_{bj}^{\xi }]=R_{ai}^{\xi }$$ 16

where ϵ_*i*_ and ϵ_*a*_ are the orbital eigenvalues of occupied orbital *i*, respectively unoccupied orbital *a*, and *G*_*ai*_[*U*_*bj*_^ξ^] denotes the auxiliary Fock matrix, here written for a hybrid functional with *c*_*x*_ exact exchange,

$$G_{ai}[U_{bj}^{\xi }]=\sum_{\mu \nu }C_{\mu a}C_{\nu i}\left(\sum_{\theta \phi }[2(\mu \nu |\theta \phi )-c_{x}(\mu \phi |\theta \nu )]U_{\theta \phi }^{\xi }+f_{\mu \nu }^{\mathrm{xc}}[U_{\theta \phi }^{\xi }]\right)$$ 17

where *U*_θϕ_^ξ^ = ∑_*bj*_ *C*_θ*b*_ *U*_*bj*_^ξ^ *C*_ϕ*j*_.

The right-hand side of the CPHF/CPKS equations depends on the first order geometric derivatives of the Fock (*F*_*ai*_^(ξ)^) and overlap matrices (*S*_*ai*_^(ξ)^)^53^

$$R_{ai}^{\xi }=F_{ai}^{\left(\xi \right)}-\epsilon_{i}S_{ai}^{\left(\xi \right)}-G_{ai}[S_{ai}^{\left(\xi \right)}]$$ 18

where the superscript (ξ) denotes partial derivatives.

Eq 16 has the same form as a linear response equation and can make use of the same parallelization and acceleration strategies employed for linear response solvers. Here, VeloxChem features an iterative reduced-space algorithm (or subspace solver) which can handle multiple right-hand sides,^46^ as is the case for the Hessian. The computationally intensive task of constructing auxiliary Fock matrices is accelerated through a GPU implementation of linear response theory.^47^ In this implementation, ERIs are evaluated using unrolled Obara–Saika recurrence relations,^48^ and the Coulomb and exchange contributions to the auxiliary Fock matrices are computed separately to maximize data locality on GPUs.^55^ The XC contributions are evaluated using a matrix multiplication based algorithm^56^ that is also accelerated on GPUs.

### Performance and Scaling

We report here the performance of our implementation of the Hessian in terms of scaling with respect to system size. The performance tests were carried out at the HF level on four systems of spherical water clusters retrieved from www.ergoscf.org. Figure 3 presents the scaling of wall time with respect to the number of contracted basis functions. In addition to the scaling of the total wall time for calculating the Hessian (green), the scaling of three individual steps of the procedure is reported: constructing the right-hand-side (RHS) for the coupled-perturbed Hartree–Fock (CPHF) equations (orange), solving the CPHF equations (purple), and calculating all terms involving second geometric derivatives (blue). For the total wall time, we observe a scaling of *O*(*N* ^3.43^) where *N* is the number of basis functions. Similar scaling is observed for the three individual steps. It is evident from the graph that the iterative procedure of solving the CPHF equations constitutes the bottleneck of the implementation.

**Figure 3:**
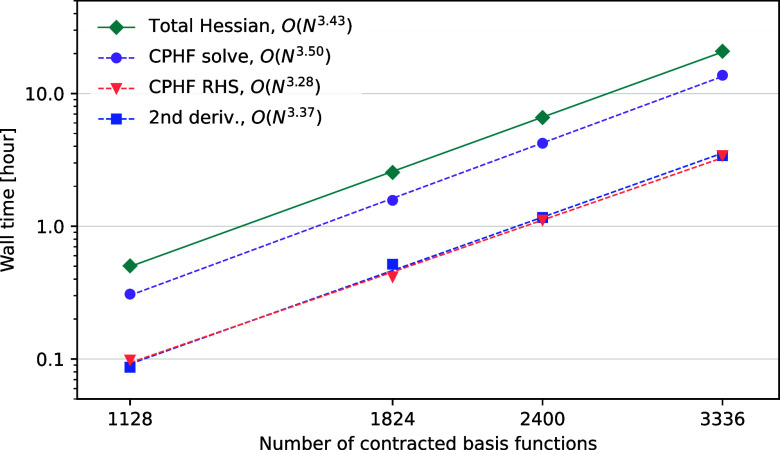
System size scaling of the total Hessian calculation and the three most time-consuming individual steps illustrated by the wall times in hours on 16 LUMI-CPU compute nodes obtained for spherical water clusters of different sizes. Each compute node is equipped with two AMD EPYC 7763 64-core processors. Calculations were performed at the HF level of theory.

#### Hybrid CPU and GPU Execution

The calculation of analytic Hessians becomescomputationally demanding as the system size increases. For a system like ubiquitin that contains 1152 atoms it is therefore very challenging to complete the whole Hessian calculation in a single job run. Instead, we chose to split the Hessian calculation of ubiquitin into smaller tasks, such that the atom-wise calculations such as evaluating the derivatives of the Fock and overlap matrices, computing the CPKS right-hand side and solving the CPKS equations can be carried out completely independently. In particular, the CPKS equations were solved using the GPU version of VeloxChem for which we implemented a subspace solver that is very similar to that used for linear response. Calculations involving the evaluation of geometric derivatives, on the other hand, were carried out using the CPU version of VeloxChem. After completion of all individual calculations, the full Hessian was assembled following the approach presented by Deglmann et al.^53^

## Application

### Computational Details

Ubiquitin is a small protein (76 amino acids) with many diverse functions in eukaryotic cells where it performs post-translational modifications of proteins.^57–59^ The most understood (and first discovered) role of this ”ubiquitination” modification is in protein degradation where the conjugation of a polyubiquitin chain targets a protein for degradation by the proteasome.^57,60,61^ Ubiquitin has a signature fold in its tertiary structure: the so-called β-grasp which consists of a five-stranded β-sheet wrapped around a central α-helix (Figure 4a).^62^

**Figure 4:**
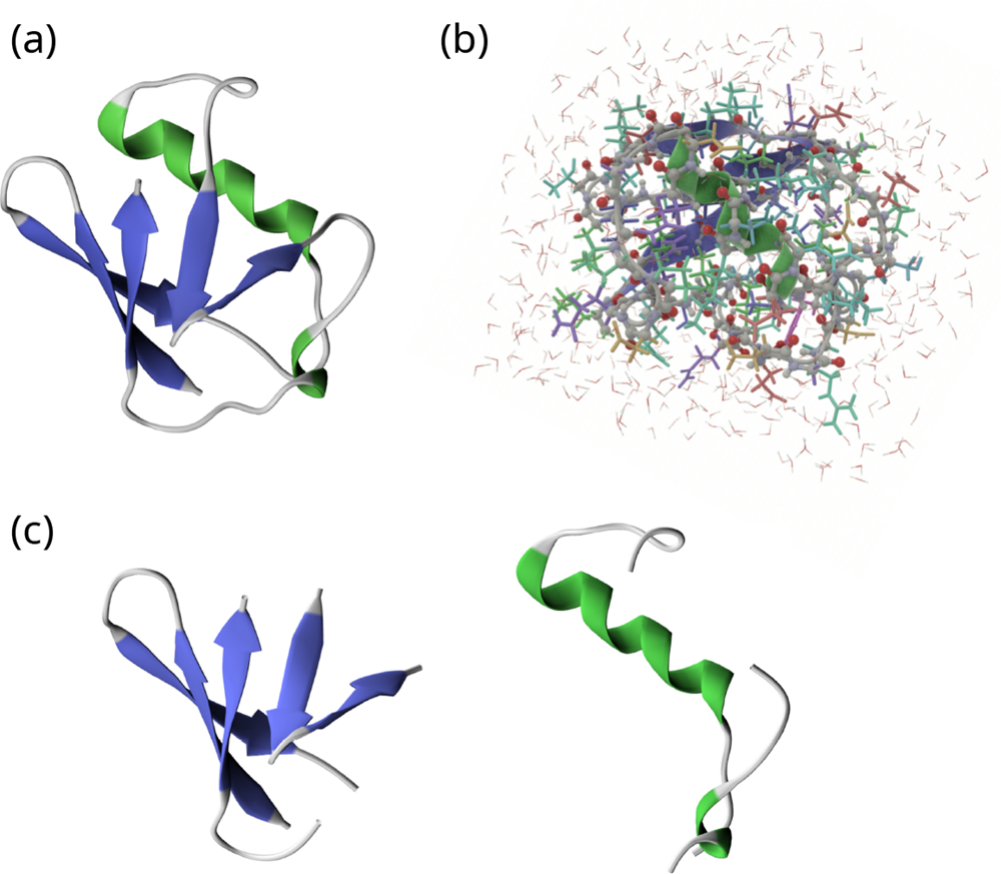
Molecular structure illustrations: (a) the tertiary structure of ubiquitin; (b) the model system for the QM/MM geometry optimization and Hessian calculation; and (c) the α (right) and β (left) subsystems for the partial Hessian approximation. Illustrations (a) and (c) were created with VMD,^70^ while (b) was made in VIAMD.^71^ Illustrations based on the MD-equilibrated structure of ubiquitin from PDB entry 1YIW.

The model system for our computational study was based on the crystal structure of the ubiquitin monomer with ID 1YIW^63^ in the protein data bank (PDB). 1YIW contains residues 1–71 and the modeled protein consisted of 1152 atoms including hydrogen atoms, with an overall charge of +2.

The system went through preparatory molecular mechanics (MM) optimizations and molecular dynamics (MD) simulations prior to the QM/MM calculations. For the MD simulations, the protein monomer was encapsulated in a 5 × 5 × 5 nm^3^ box of TIP3P^64^ water molecules. The overall charge of the box was neutralized by adding two sodium cations. The protein and ions were described using the AMBER ff99SB-ILDN force field.^65^ The GROMACS program^66^ was used for all MD calculations. First, the box with protein, ions, and water molecules was optimized with respect to the adopted MM force field. To optimize the density of the box, an MD simulation of 1 ns in the *NPT* ensemble was performed. The reference temperature was 300 K, maintained by the velocity rescaling weak coupling scheme^67^ with a time constant of τ = 0.1 ps. The reference pressure was 1 bar, maintained using stochastic cell rescaling^68^ with a coupling constant of τ = 2.0 ps. A subsequent *NVT* simulation was performed with a duration of 2 ns using the same thermostat as for the *NVT* ensemble. For all MD simulations, the time step was 2 fs and electrostatic interactions were modeled with the particle mesh Ewald (PME) approach^69^ with a cutoff distance of 1.0 nm. A position constraint of 1000 kJ·mol^–1^·nm^–2^ was applied to restrict the protein backbone. The final snapshot structure of the *NVT* simulation was optimized under the adopted force field, and this MM optimized system was used as the starting point for building the model system for the QM/MM geometry optimization and vibrational analysis.

The QM/MM model system consisted of the protein and water molecules within a 5 Å cutoff, see Figure 4b. No sodium ions were present within this region, and thus the overall charge was +2. For the QM/MM geometry optimization, the interactions between the QM and MM regions were modeled with electrostatic embedding. The QM region was defined by the protein and described with the def2-SV­(P) basis set and the MM region was a water solvation shell with TIP3P point charges (−0.834 for O and 0.417 for H). The QM/MM optimization was carried out on the charged system. Dispersion was included for some optimization steps to assess its effect but it proved to have very little influence on the gradient. The inclusion of dispersion was then discontinued. The SCF equations were converged under a threshold of 10^–6^ with an ERI screening threshold of 10^–12^ and a molecular grid level of 4. When the molecular structure of the protein was converged to a satisfactory threshold, the molecular Hessian was calculated at the B3LYP/def2-SV­(P) level of theory.

All quantum chemical calculations were carried out with the VeloxChem program.^46,47^ The VIAMD software^71^ was used for the visual analysis of the vibrational modes of the protein.

To investigate the effect of diagonalizing only parts of the molecular Hessian, the protein was split into two subsystems separating the α-helices and β-sheet. According to the PDB entry for 1YIW there are β-strands between residues 2–6, 12–16, 43–45, 48–49, and 66–69, while three subsequences of α-helices are found between residues 22–35, 37–41, 56–60. The protein was cut into fragments between GLU18/PRO19, GLN41/ARG42, GLU51/ASP52, and ASN60/ILE61 such that the α-subsystem contained PRO19–GLN41 and ASP52–ASN60 (488 atoms) while the fragments LEU1–GLU18, GLN41–GLU51, and ILE61–LEU71 made up the β-subsystem (664 atoms). The two model subsystems are illustrated in Figure 4c. The computational process was then to extract the respective fragments of the full Hessian and carry out separate vibrational analyses. As we have here access to the Hessian for the full protein, there is no computational saving (other than matrix diagonalizations) associated with the subsystem approach. In a real use case scenario, on the other hand, one would want to avoid the calculation of the Hessian of the full system and rather perform separate Hessian calculations for the subsystems capped in ways as to add as few atoms as possible while retaining the true electronic structures found in the full system. Our investigation removes this issue of cap appropriateness from the discussion and thereby pinpoints the effects of the partial Hessian approximation.

In VeloxChem, the IR intensity of normal mode *k* is calculated as the Napierian integrated attenuation coefficient^72,73^

$$A_{k}=\frac{1}{4\pi \varepsilon_{0}}\frac{N_{A}\pi }{3c^{2}}|\frac{d\mu }{dQ_{k}}{|}^{2}$$ 19

where **μ** is the electric dipole moment and *Q*_*k*_ is the normal coordinate of normal mode *k*, *N*_A_ is the Avogadro constant, ε_0_ is the vacuum permittivity, and *c* is the speed of light in vacuum. The dipole moment gradient is expressed in Cartesian coordinates according to

$$\frac{d\mu }{dQ_{k}}=\sum_{i}\frac{\partial \mu }{\partial x_{i}}\frac{dx_{i}}{dQ_{k}}=\sum_{i}\frac{\partial \mu }{\partial x_{i}}l_{ik}$$ 20

where the transformation coefficients between Cartesian and normal coordinates, *l*_*ik*_, have the unit of $1/\sqrt{\mathrm{amu}}$ . To obtain the IR intensity in km/mol, one notes that |d**μ**/d*Q*_*k*_| ^2^ is in unit of *e* ^2^/amu where *e* is the elementary charge. Combining the physical constants in eq 19 with a conversion from *e* ^2^/amu to SI units and from m to km results in a final prefactor of 974.88 km/mol amu/*e* ^2^.

With attenuation coefficients available, the IR absorption spectrum is constructed as

$$\sigma (\omega )=\sum_{k}A_{k}f(\omega ;\omega_{k0},\gamma )$$ 21

where, in the present work, *f*(ω;ω_*k*0_, γ) is chosen as a Lorentzian broadening with γ being the half-width-at-half-maximum (HWHM):

$$f(\omega ;\omega_{k0},\gamma )=\frac{1}{\pi }\frac{\gamma }{(\omega_{k0}-\omega {)}^{2}+\gamma^{2}}$$ 22

Both homogeneous and inhomogeneous broadening of absorption bands occur for proteins in solution. The inhomogeneous broadening, generally described by a Gaussian line shape, arises from the conformational freedom of the molecular structure of the solute, while the short lifetime of the vibrational excited state gives bands that are generally Lorentzian.^74^ The type of broadening thus depends on the experimental details, but we have here restricted ourselves to adopting the Lorentzian line shape.

All vibrational frequencies obtained from the computational vibrational analyses were scaled by an overall common factor of 0.957. This scaling factor was determined from fitting a simulated spectrum to an experimental spectrum in the region 1720–1570 cm^–1^.

### Spectrum Simulations

The normal-mode analysis revealed 3450 vibrational modes of which 226 modes had frequencies below 100 cm^–1^ with a highest absolute value of 183 cm^–1^. Figure 5 shows the simulated IR spectrum in the frequency region of 3500–300 cm^–1^ with the protein-characteristic amide A and I–VI bands labeled in the figure. The NH stretching vibrations in the backbone structure appear at high wavenumbers between 3500–3130 cm^–1^ and are referred to as the amide A vibrations.^13^ The spectral feature (3130–2900 cm^–1^) following the amide A band covers general CH stretching modes in the molecule.

**Figure 5:**
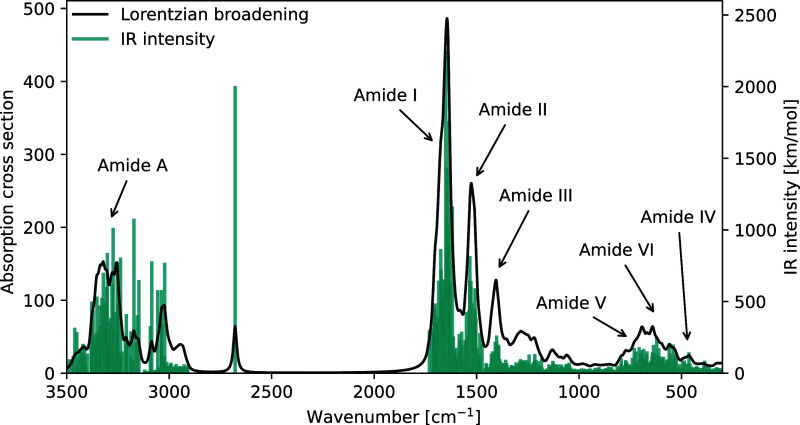
IR spectrum of ubiquitin based on the full Hessian. A Lorentzian broadening of 10 cm^–1^ (HWHM) has been applied and the vibrational frequencies are scaled by a factor of 0.957. Calculations were performed at the B3LYP/def2-SV­(P) level of theory.

Three distinct peaks appear in the midrange frequency region, constituting the amide I (1730–1620 cm^–1^), amide II (1620–1470 cm^–1^), and amide III (1470–1340 cm^–1^) bands. The amide I band arises from the backbone CO stretching vibration, amide II from the out-of-phase combination of the backbone NH in-plane bend and CN stretch, while amide III is the in-phase combination of the NH bending and CN stretching vibrations.^13,16^ At lower wave numbers, the amide V band is identified between 820–660 cm^–1^, amide VI between 660–580 cm^–1^, and the amide IV between 580–510 cm^–1^.

The simulated IR spectrum in the amide I region is shown in Figure 6 alongside the experimental spectrum from ref 75. There is excellent agreement between the experiment and our computational results. Both spectra are left skewed with several shoulders at higher wavenumbers and one less pronounced shoulder toward lower wavenumbers.

**Figure 6:**
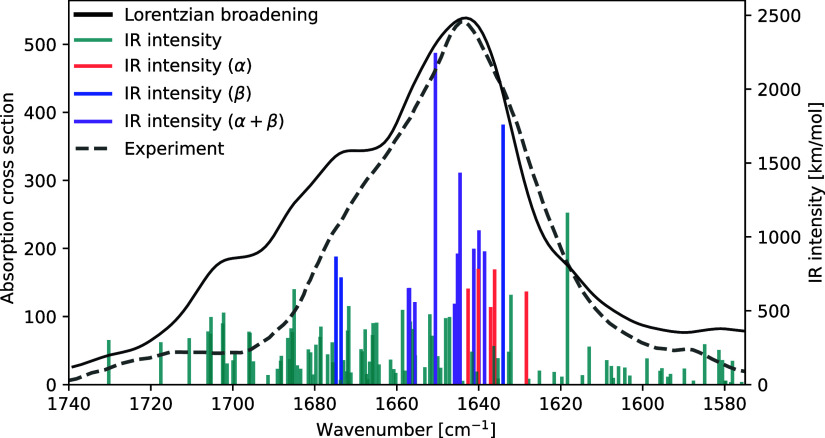
IR spectrum of ubiquitin in the amide I region. Transitions stronger than 500 km/mol are color coded according to mode localizations in α-helices (orange), β-sheets (blue), or both (purple). Intense transitions with modes localized in turns, coils, and/or side chains are not color coded. Lorentzian broadening of 7.5 cm^–1^ (HWHM), vibrational frequencies scaled by a factor of 0.957. Calculations were performed at the B3LYP/def2-SV­(P) level of theory. Experimental spectrum from ref 75.

The localization of vibrational modes within the molecular structure has been determined by visual inspection using VIAMD. The coloring of the IR intensity sticks in the spectrum refers to the structural localization of the vibrations for transitions stronger than 500 km/mol. The first two transitions seen at higher wavenumbers in Figure 6 are related to CO stretching vibrations in side chain amide groups. In the region 1710–1680 cm^–1^, the CO vibrations are predominantly located in the coils and turns of the backbone, with some contributions from the α-helices.

Investigating the transitions with intensities of 500 km/mol or more (color coded), it is seen that vibrations in the β structures (blue) give rise to a very intense mode at 1634 cm^–1^ and two less intense modes at 1675 and 1674 cm^–1^. This is in agreement with the rule-of-thumb that antiparallel β sheets exhibit a main band around 1630 cm^–1^ with a weaker band around 1685 cm^–1^.^13^ Meanwhile, four vibrational modes mainly located in the α helices (orange) are identified between 1643 and 1636 cm^–1^ with a fifth mode at 1628 cm^–1^. The general spectral features of α-helix secondary structures are a main band at approximately 1650 cm^–1^ with a shoulder at lower wavenumbers.^13^ However, an α-helix forming hydrogen bonds to solvent molecules may exhibit a main absorption as much as 20 cm^–1^ lower.^12^ It is reasonable to assume that the QM/MM solvent embedding applied in our calculations is able to exert this effect on the protein, shifting the main α band toward 1640 cm^–1^. Vibrational modes that are pronounced in both secondary structures (but absent in coils and turns) are drawn in purple and observed at frequencies between 1657 and 1639 cm^–1^, including the most IR intense mode at 1651 cm^–1^. The last amide I-type vibration is identified at 1628 cm^–1^.

#### Spectrum Simulations with Partial Hessians

IR spectra for the α and β subsystems, as well as the sum of the two, are presented across the entire IR region in Figure 7 and in the amide I region in Figure 8. In the latter, color coding of the IR intensities is employed according to the same scheme as in Figure 6. Both subsystems exhibit the characteristic amide bands identified previously for the full Hessian spectrum. The single intense mode around 2680 cm^–1^ observed in the β-subsystem spectrum (Figure 7b) is the same as the one observed in the IR spectrum calculated from the full Hessian (Figure 5). At a first glance, one can hardly detect the difference between the IR spectrum in Figure 5 and the combined spectra of the two subsystems in Figure 7c.

**Figure 7:**
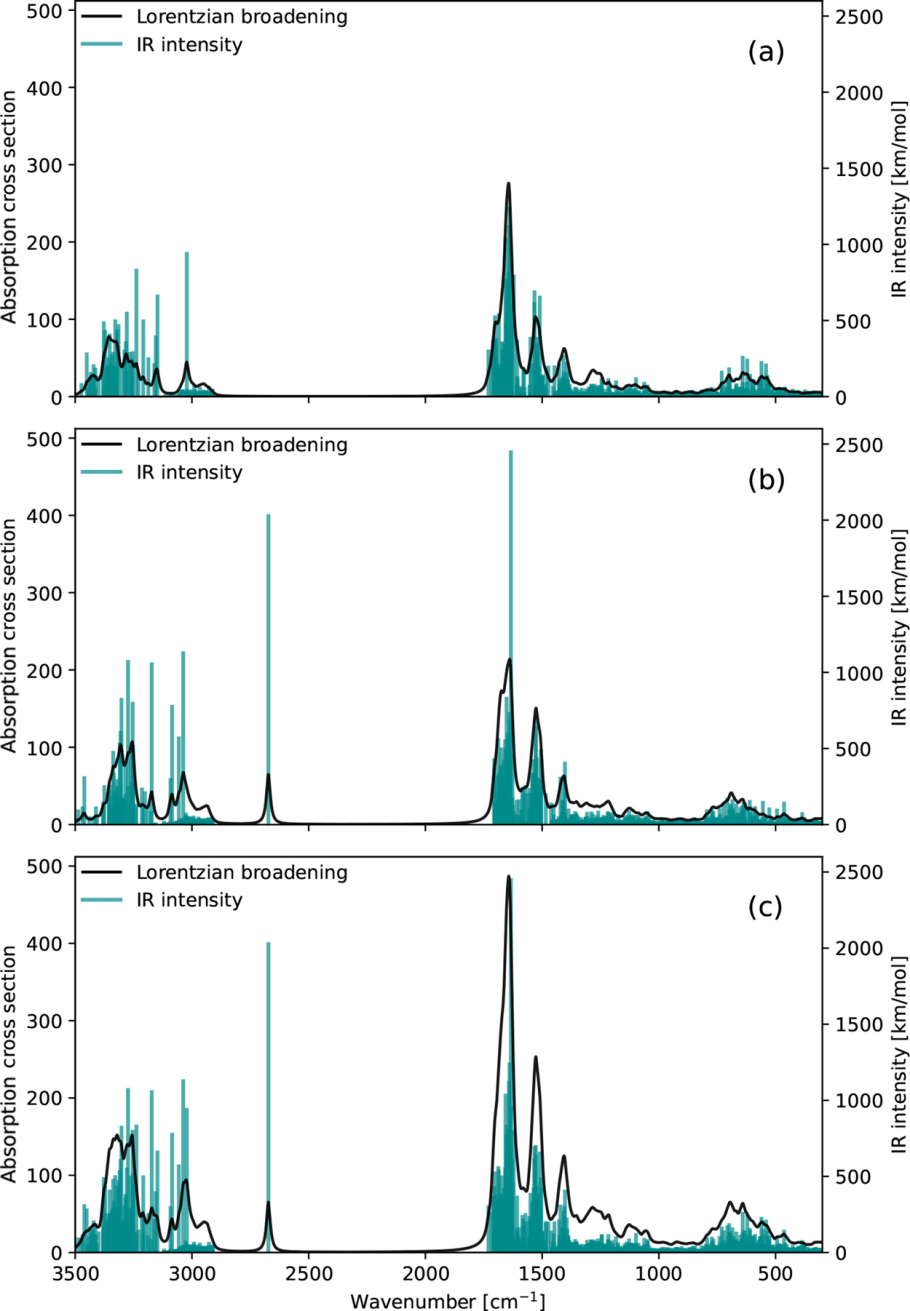
IR spectra of the (a) α-subsystem and (b) β-subsystem. Panel (c) shows the sum of the two. Lorentzian broadening of 10 cm^–1^ (HWHM), vibrational frequencies scaled by a factor of 0.957. Calculations were performed at the B3LYP/def2-SV­(P) level of theory.

**Figure 8:**
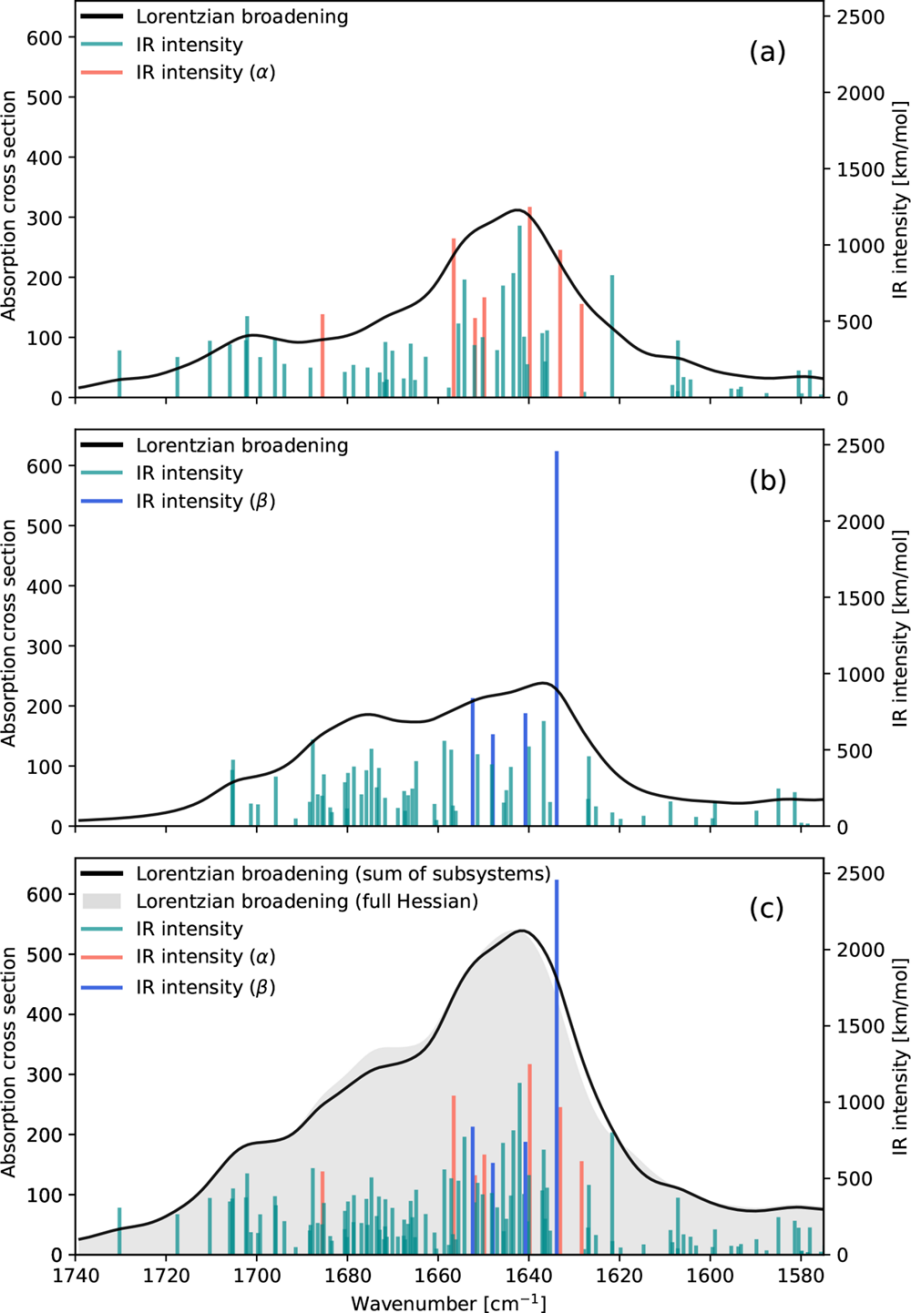
Amide I region IR spectra of (a) subsystem containing α-helix secondary structures; (b) subsystem containing β-sheet secondary structures; and (c) combined spectrum for the α and β subsystems. Lorentzian broadening of 7.5 cm^–1^ (HWHM), vibrational frequencies scaled by a factor of 0.957. Calculations were performed at the B3LYP/def2-SV­(P) level of theory.

Visual inspection of the 12 vibrational modes in the α-subsystem with an intensity higher than 500 km/mol reveals seven modes mainly localized in one or more of the α-helices, see Figure 8a. Except for one localized transition at 1685 cm^–1^, most vibrate at frequencies in the range 1657–1628 cm^–1^. This is in agreement with the amide I spectrum obtained from the full Hessian, where this range contained all “α” and “α + β” modes. It is noted that in the full Hessian spectrum the pure α-helix vibrations were all below 1643 cm^–1^, while in the partial Hessian spectrum, such vibrations are now also present in the frequency range that was previously dominated by modes involving both types of secondary structure. The mode at 1685 cm^–1^ is within the range that was previously determined to mainly convolute vibrations in coils with some influence of α-helix amide I vibrations. In general we note that the frequency interval where intense modes were assigned to be “α + β”, the subsystems have vibrational modes localized in their respective type of secondary structure.

Only eight transitions were above the 500 km/mol threshold for visual inspection, and only four were characterized as being localized in the β-sheet. The one at 1634 cm^–1^ has a very high intensity and seems to correspond to the β-localized transition at the same frequency in Figure 5. Comparing to the full Hessian amide I spectrum, it appears that the two β-localized modes at higher wavenumbers have disappeared, since similar modes are not included in the set of most intense transitions in the β-subsystem spectrum. However, there are still two modes localized in the β-sheet at 1675 and 1674 cm^–1^, only with intensities of 494 and 469 km/mol in the β subsystem, respectively.

Figure 8c shows the amide I IR spectrum obtained by combining the spectra for the two subsystems, with the full Hessian spectrum shown as gray fill. We see that the sum of the subsystems reproduces it fairly well.

## Conclusions and Outlook

We have successfully carried out a fully analytical first-principles calculation of the Hessian of the ubiquitin protein consisting of 1152 atoms. This endeavor was made possible by a highly efficient implementation of geometric derivatives up to second order of ERIs in the VeloxChem software, targeting execution in modern HPC environments with hybrid usage of CPU and GPU resources.

Our calculations revealed results of exceptional quality, reproducing the experimental amide I spectrum of ubiquitin. A visual analysis confirmed that the spectral features observed in the full IR spectrum correspond to the different amide bands characteristic for proteins. Furthermore, vibrational modes localized on the different types of secondary structures could be identified and connected to rule-of-thumb spectral shapes of such structures.

Furthermore, we investigated the effects of the partial Hessian approximation by splitting the protein into two subsystems, each containing either α-helix or β-sheet structures. This approach neglects the coupling terms between the two subsystem in the diagonalization of the Hessian. Nevertheless, when adding together the IR spectra of the subsystems, the result was a more than fair reproduction of the spectrum obtained from diagonalizing the full Hessian. This result speaks in support of the partial Hessian approach, however, keeping in mind that we introduced the approximation after having computed the electronic structure and Hessian of the entire protein. A practical employment of the partial Hessian approximation also involves the computational consideration of capping the fragments.

With interest, we also note the single-point Hessian method^76^ that, agnostic about the underlying electronic structure theory, allows for the calculation of IR spectra at arbitrary molecular structures that not necessarily represent stationary points on the potential energy surface and which may be useful for the treatment of complex systems where a room temperature molecular dynamics (MD) sampling is called for.
